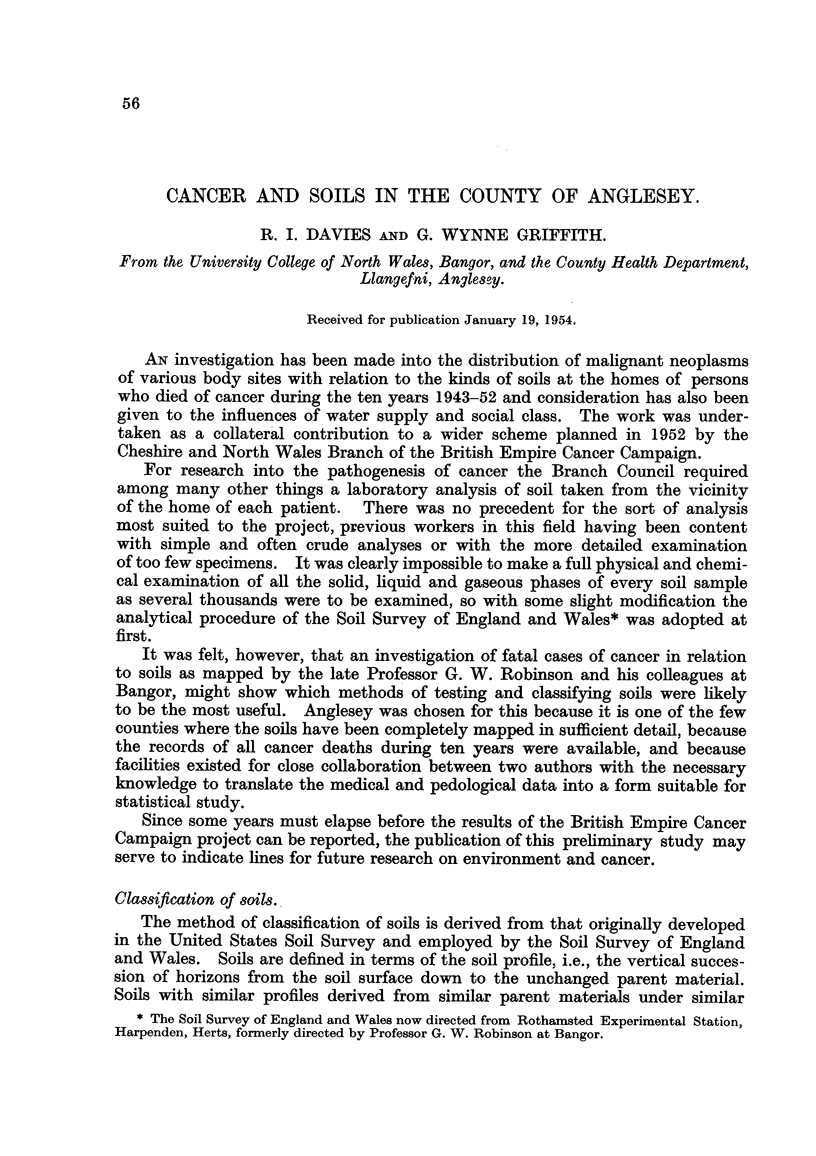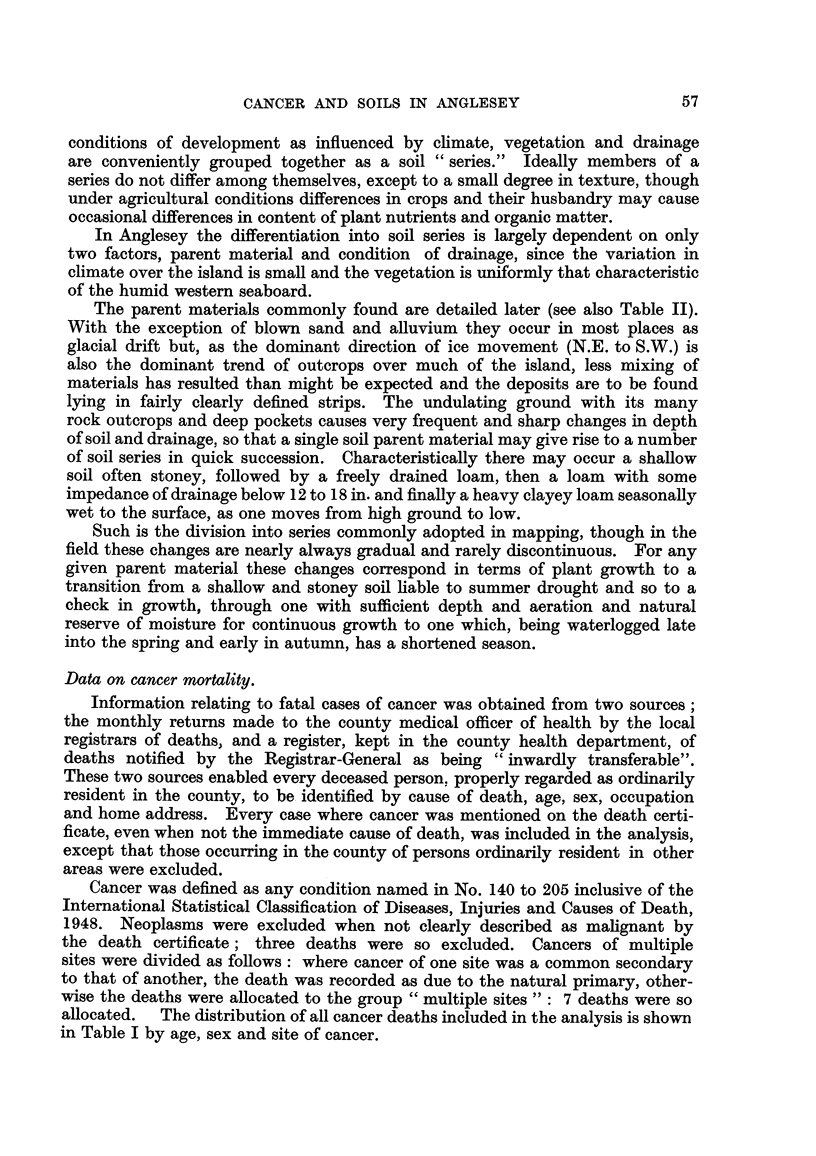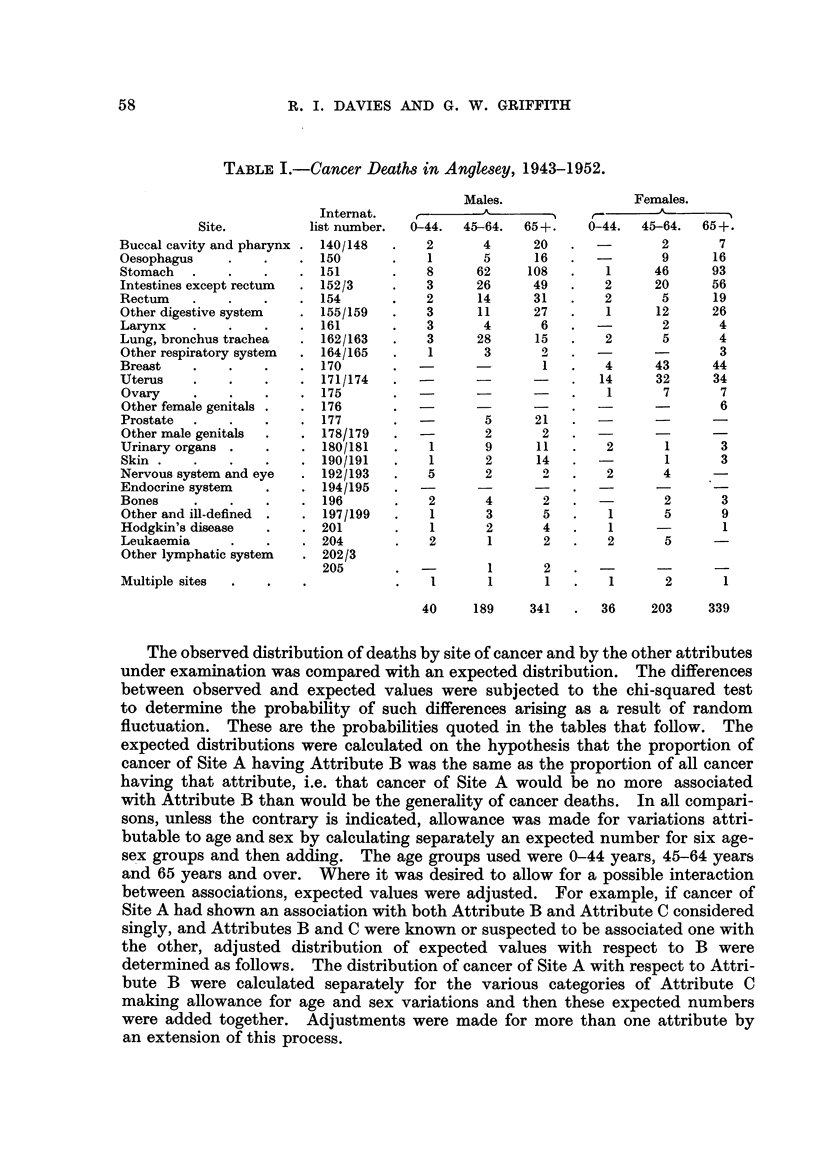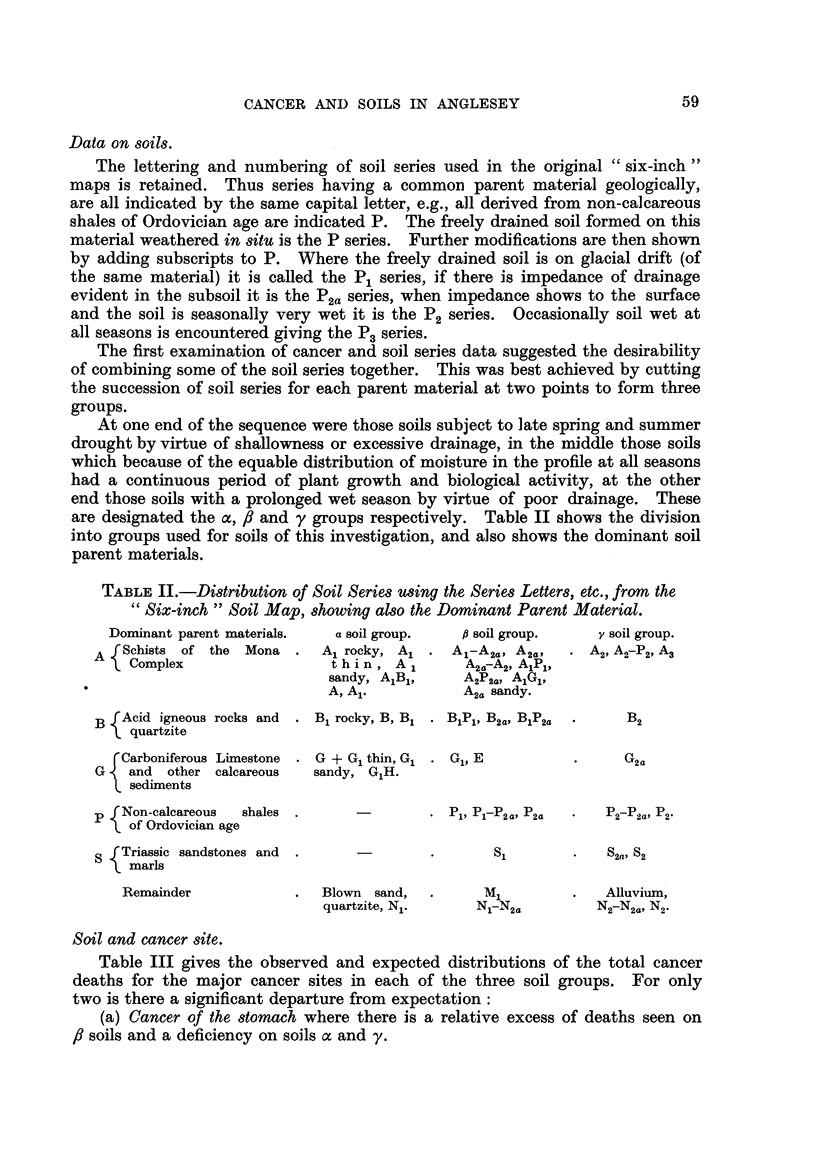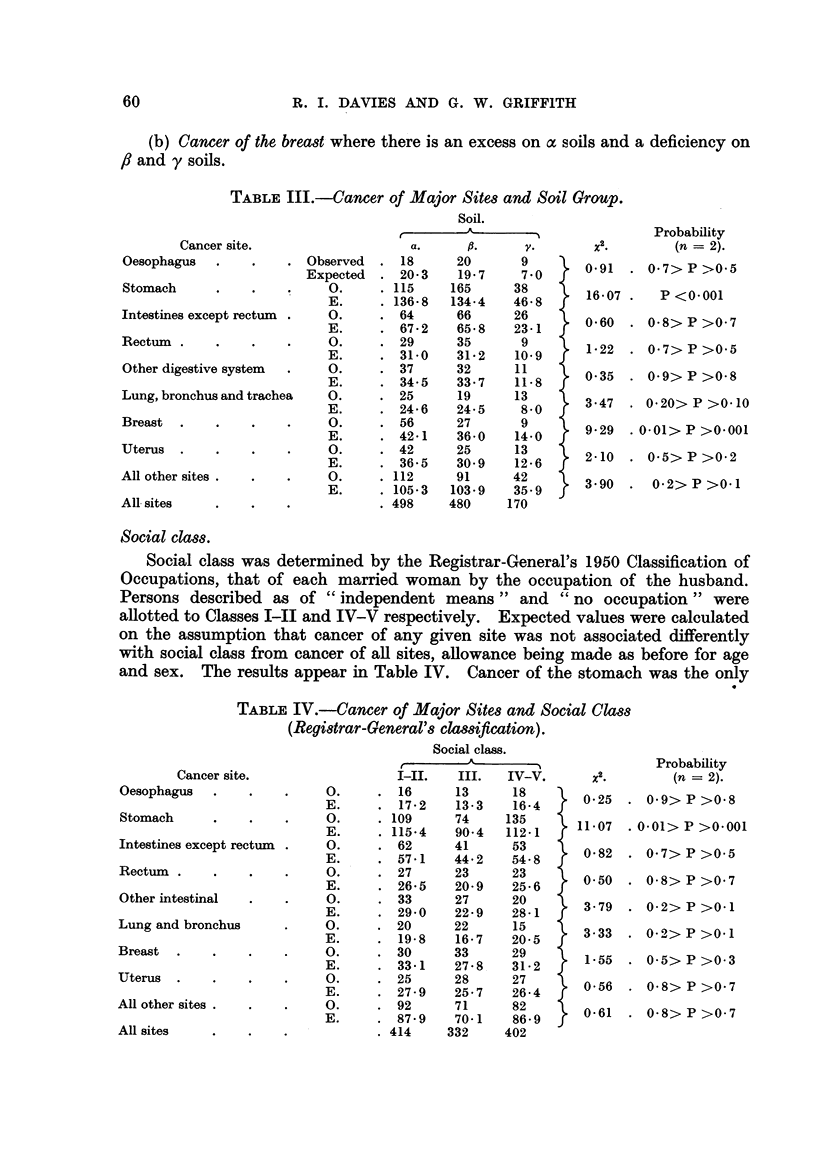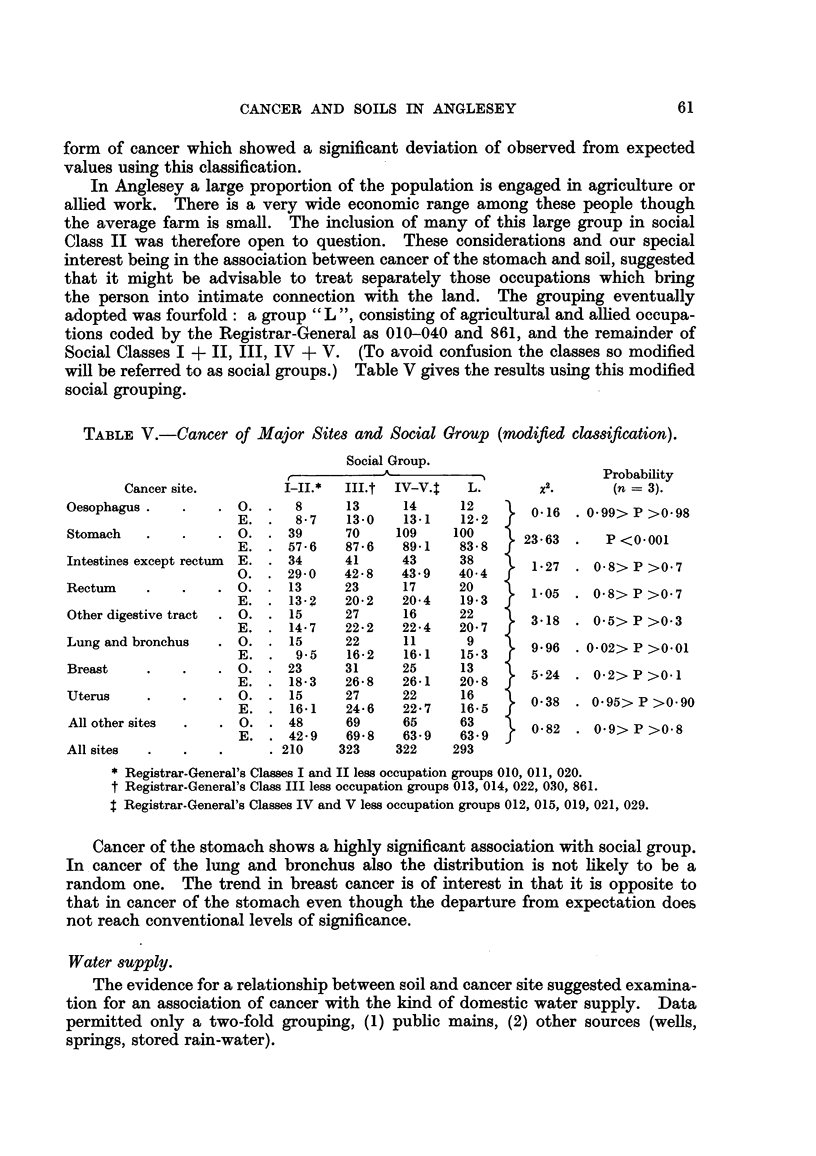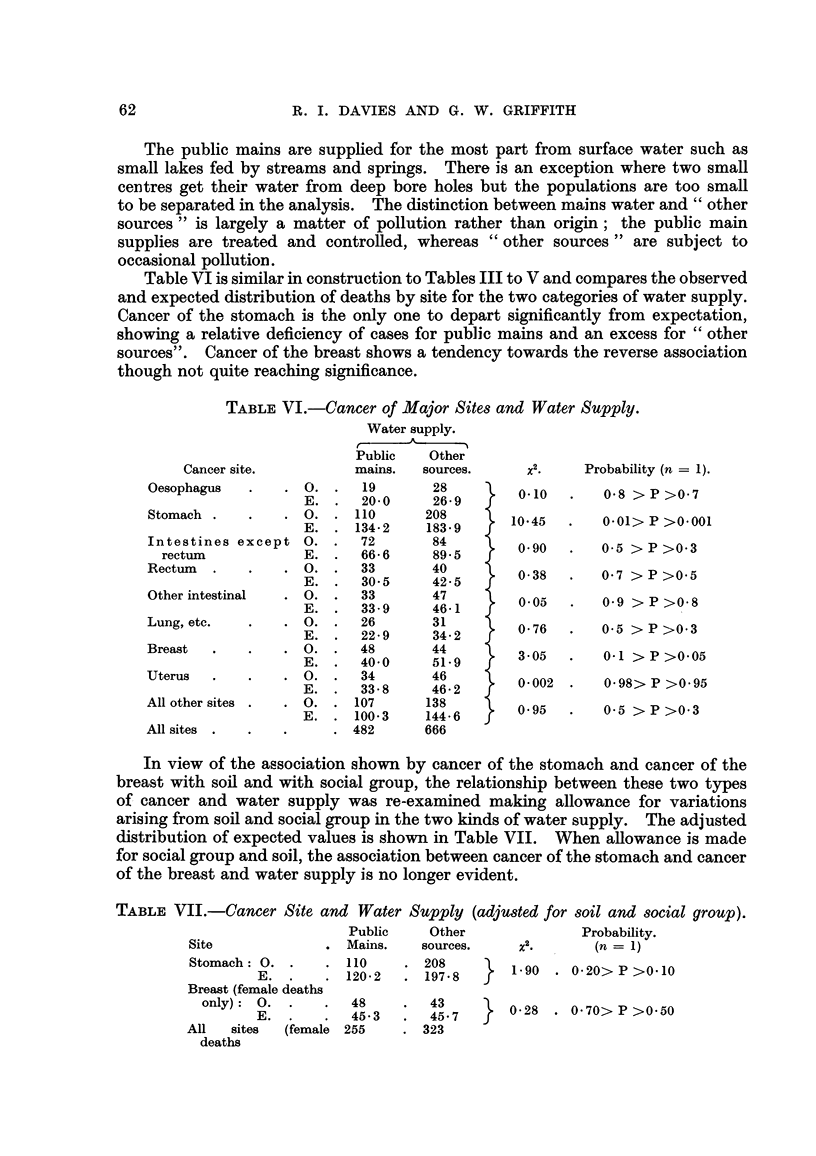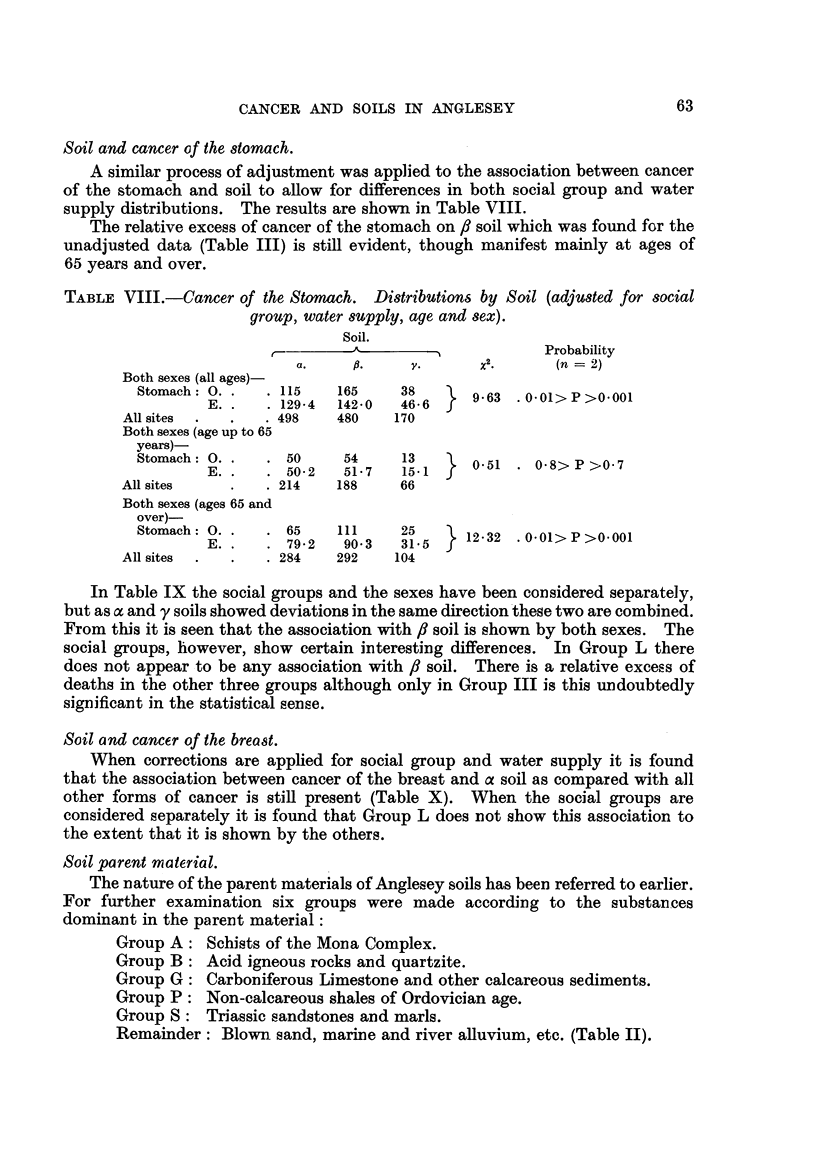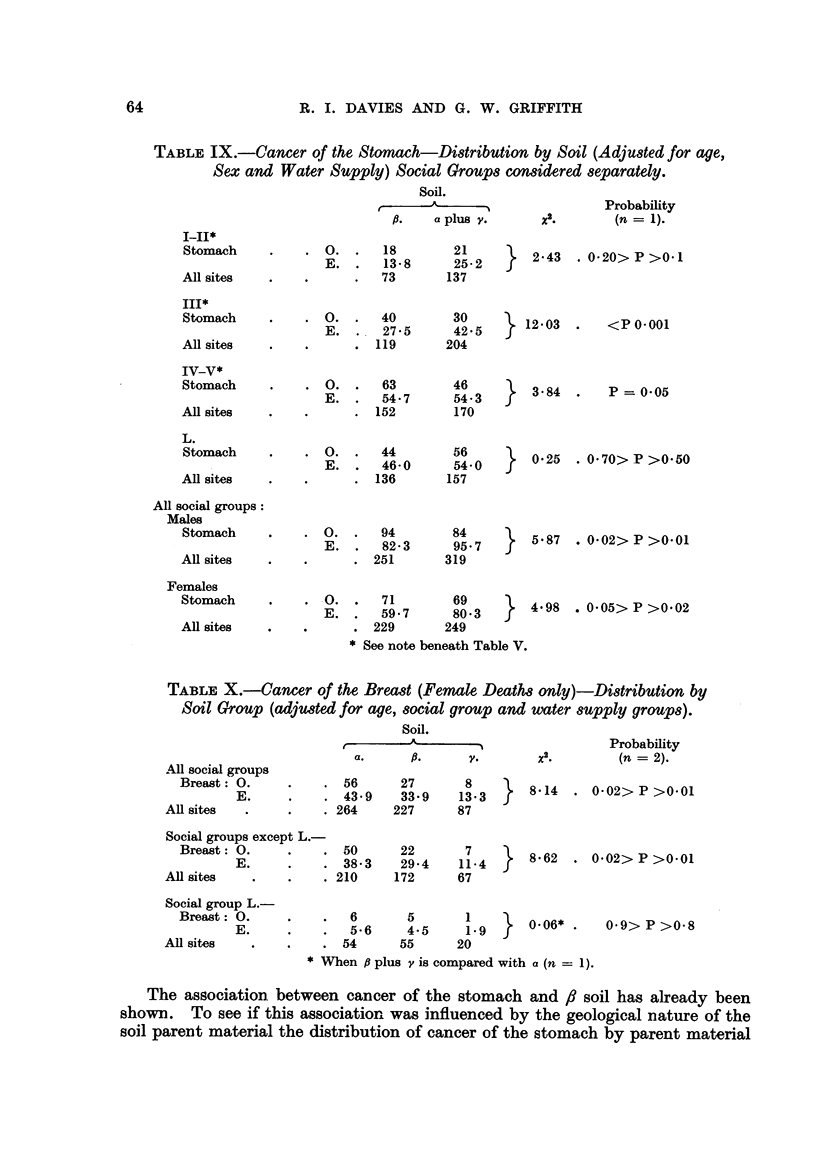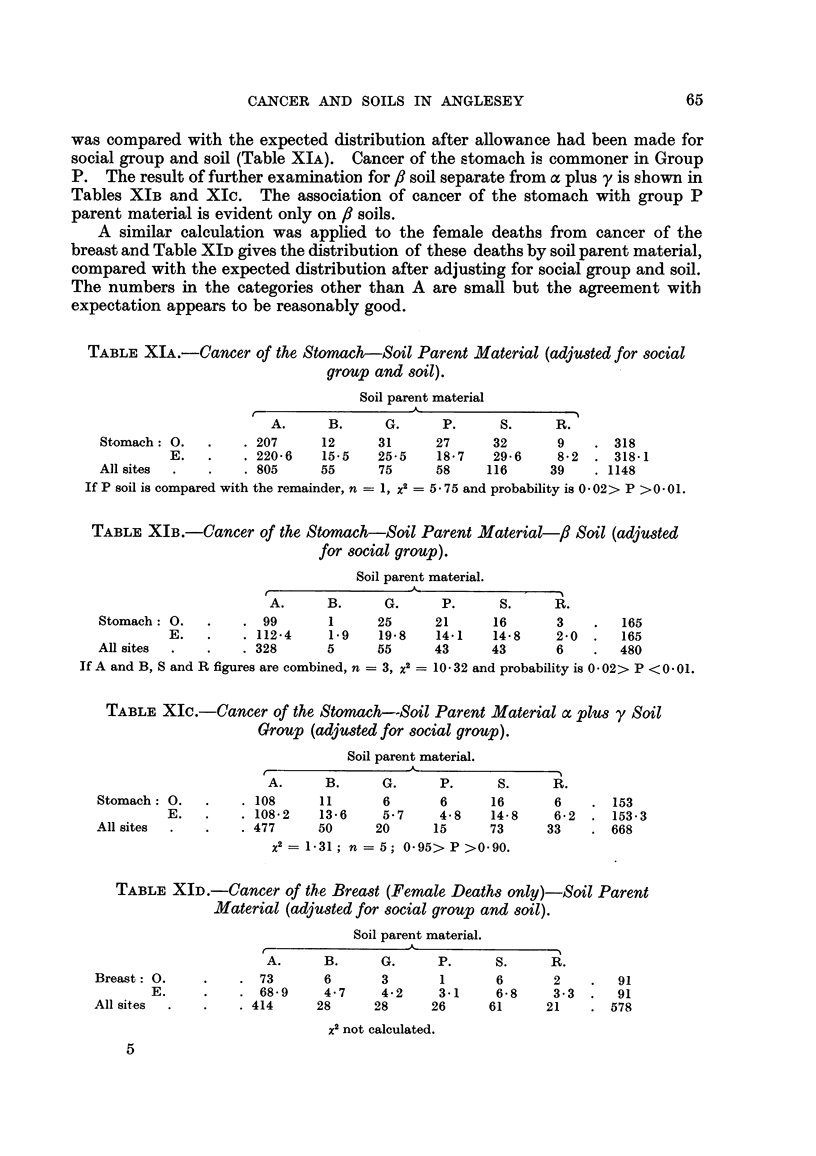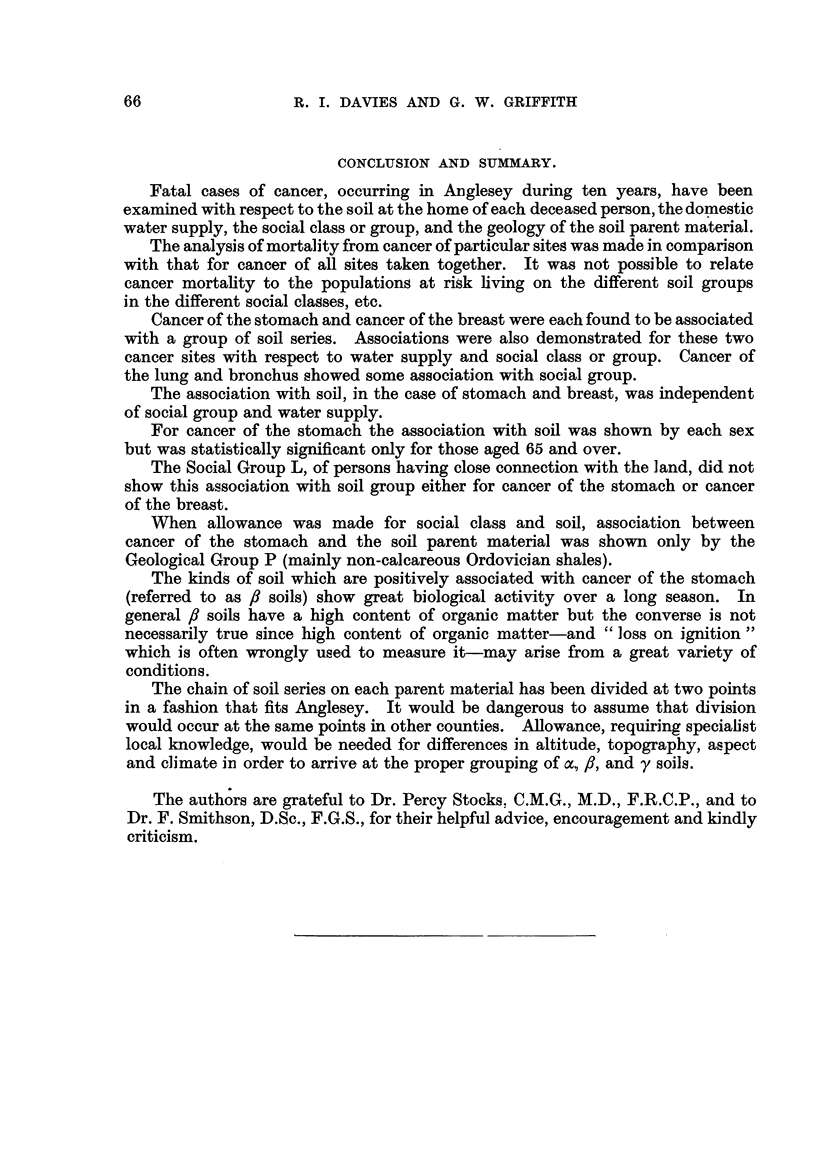# Cancer and Soils in the County of Anglesey

**DOI:** 10.1038/bjc.1954.5

**Published:** 1954-03

**Authors:** R. I. Davies, G. Wynne Griffith


### CANCER AND SOILS IN THE COUNTY OF ANGLESEY.

R. I. DAVIES AND G. WYNNE GRIFFITH.

*From the University College of North Wales, Bangor, and the County Health Department, Llangefni, Anglesey.*

Received for publication January 19, 1954.

AN investigation has been made into the distribution of malignant neoplasms of various body sites with relation to the kinds of soils at the homes of persons who died of cancer during the ten years 1943–52 and consideration has also been given to the influences of water supply and social class. The work was undertaken as a collateral contribution to a wider scheme planned in 1952 by the Cheshire and North Wales Branch of the British Empire Cancer Campaign.

For research into the pathogenesis of cancer the Branch Council required among many other things a laboratory analysis of soil taken from the vicinity of the home of each patient. There was no precedent for the sort of analysis most suited to the project, previous workers in this field having been content with simple and often crude analyses or with the more detailed examination of too few specimens. It was clearly impossible to make a full physical and chemical examination of all the solid, liquid and gaseous phases of every soil sample as several thousands were to be examined, so with some slight modification the analytical procedure of the Soil Survey of England and Wales* was adopted at first.

It was felt, however, that an investigation of fatal cases of cancer in relation to soils as mapped by the late Professor G. W. Robinson and his colleagues at Bangor, might show which methods of testing and classifying soils were likely to be the most useful. Anglesey was chosen for this because it is one of the few counties where the soils have been completely mapped in sufficient detail, because the records of all cancer deaths during ten years were available, and because facilities existed for close collaboration between two authors with the necessary knowledge to translate the medical and pedological data into a form suitable for statistical study.

Since some years must elapse before the results of the British Empire Cancer Campaign project can be reported, the publication of this preliminary study may serve to indicate lines for future research on environment and cancer.

### *Classification of soils.*

The method of classification of soils is derived from that originally developed in the United States Soil Survey and employed by the Soil Survey of England and Wales. Soils are defined in terms of the soil profile, i.e., the vertical succession of horizons from the soil surface down to the unchanged parent material. Soils with similar profiles derived from similar parent materials under similar

* The Soil Survey of England and Wales now directed from Rothamsted Experimental Station, Harpenden, Herts, formerly directed by Professor G. W. Robinson at Bangor.

conditions of development as influenced by climate, vegetation and drainage are conveniently grouped together as a soil " series." Ideally members of a series do not differ among themselves, except to a small degree in texture, though under agricultural conditions differences in crops and their husbandry may cause occasional differences in content of plant nutrients and organic matter.

In Anglesey the differentiation into soil series is largely dependent on only two factors, parent material and condition of drainage, since the variation in climate over the island is small and the vegetation is uniformly that characteristic of the humid western seaboard.

The parent materials commonly found are detailed later (see also Table II). With the exception of blown sand and alluvium they occur in most places as glacial drift but, as the dominant direction of ice movement (N.E. to S.W.) is also the dominant trend of outcrops over much of the island, less mixing of materials has resulted than might be expected and the deposits are to be found lying in fairly clearly defined strips. The undulating ground with its many rock outcrops and deep pockets causes very frequent and sharp changes in depth of soil and drainage, so that a single soil parent material may give rise to a number of soil series in quick succession. Characteristically there may occur a shallow soil often stoney, followed by a freely drained loam, then a loam with some impedance of drainage below 12 to 18 in. and finally a heavy clayey loam seasonally wet to the surface, as one moves from high ground to low.

Such is the division into series commonly adopted in mapping, though in the field these changes are nearly always gradual and rarely discontinuous. For any given parent material these changes correspond in terms of plant growth to a transition from a shallow and stoney soil liable to summer drought and so to a check in growth, through one with sufficient depth and aeration and natural reserve of moisture for continuous growth to one which, being waterlogged late into the spring and early in autumn, has a shortened season.

*Data on cancer mortality.*

Information relating to fatal cases of cancer was obtained from two sources ; the monthly returns made to the county medical officer of health by the local registrars of deaths, and a register, kept in the county health department, of deaths notified by the Registrar-General as being " inwardly transferable". These two sources enabled every deceased person, properly regarded as ordinarily resident in the county, to be identified by cause of death, age, sex, occupation and home address. Every case where cancer was mentioned on the death certificate, even when not the immediate cause of death, was included in the analysis, except that those occurring in the county of persons ordinarily resident in other areas were excluded.

Cancer was defined as any condition named in No. 140 to 205 inclusive of the International Statistical Classification of Diseases, Injuries and Causes of Death, 1948. Neoplasms were excluded when not clearly described as malignant by the death certificate ; three deaths were so excluded. Cancers of multiple sites were divided as follows : where cancer of one site was a common secondary to that of another, the death was recorded as due to the natural primary, otherwise the deaths were allocated to the group " multiple sites " : 7 deaths were so allocated. The distribution of all cancer deaths included in the analysis is shown in Table I by age, sex and site of cancer.

TABLE I.—*Cancer Deaths in Anglesey*, 1943–1952.

| Site. | Internat. list number. | Males. 0–44. | Males. 45–64. | Males. 65+. | Females. 0–44. | Females. 45–64. | Females. 65+. |
|---|---|---|---|---|---|---|---|
| Buccal cavity and pharynx | 140/148 | 2 | 4 | 20 | — | 2 | 7 |
| Oesophagus | 150 | 1 | 5 | 16 | — | 9 | 16 |
| Stomach | 151 | 8 | 62 | 108 | 1 | 46 | 93 |
| Intestines except rectum | 152/3 | 3 | 26 | 49 | 2 | 20 | 56 |
| Rectum | 154 | 2 | 14 | 31 | 2 | 5 | 19 |
| Other digestive system | 155/159 | 3 | 11 | 27 | 1 | 12 | 26 |
| Larynx | 161 | 3 | 4 | 6 | — | 2 | 4 |
| Lung, bronchus trachea | 162/163 | 3 | 28 | 15 | 2 | 5 | 4 |
| Other respiratory system | 164/165 | 1 | 3 | 2 | — | — | 3 |
| Breast | 170 | — | — | 1 | 4 | 43 | 44 |
| Uterus | 171/174 | — | — | — | 14 | 32 | 34 |
| Ovary | 175 | — | — | — | 1 | 7 | 7 |
| Other female genitals | 176 | — | — | — | — | — | 6 |
| Prostate | 177 | — | 5 | 21 | — | — | — |
| Other male genitals | 178/179 | — | 2 | 2 | — | — | — |
| Urinary organs | 180/181 | 1 | 9 | 11 | 2 | 1 | 3 |
| Skin | 190/191 | 1 | 2 | 14 | — | 1 | 3 |
| Nervous system and eye | 192/193 | 5 | 2 | 2 | 2 | 4 | — |
| Endocrine system | 194/195 | — | — | — | — | — | — |
| Bones | 196 | 2 | 4 | 2 | — | 2 | 3 |
| Other and ill-defined | 197/199 | 1 | 3 | 5 | 1 | 5 | 9 |
| Hodgkin's disease | 201 | 1 | 2 | 4 | 1 | — | 1 |
| Leukaemia | 204 | 2 | 1 | 2 | 2 | 5 | — |
| Other lymphatic system | 202/3 205 | — | 1 | 2 | — | — | — |
| Multiple sites | | 1 | 1 | 1 | 1 | 2 | 1 |
| | | 40 | 189 | 341 | 36 | 203 | 339 |

The observed distribution of deaths by site of cancer and by the other attributes under examination was compared with an expected distribution. The differences between observed and expected values were subjected to the chi-squared test to determine the probability of such differences arising as a result of random fluctuation. These are the probabilities quoted in the tables that follow. The expected distributions were calculated on the hypothesis that the proportion of cancer of Site A having Attribute B was the same as the proportion of all cancer having that attribute, i.e. that cancer of Site A would be no more associated with Attribute B than would be the generality of cancer deaths. In all comparisons, unless the contrary is indicated, allowance was made for variations attributable to age and sex by calculating separately an expected number for six age-sex groups and then adding. The age groups used were 0–44 years, 45–64 years and 65 years and over. Where it was desired to allow for a possible interaction between associations, expected values were adjusted. For example, if cancer of Site A had shown an association with both Attribute B and Attribute C considered singly, and Attributes B and C were known or suspected to be associated one with the other, adjusted distribution of expected values with respect to B were determined as follows. The distribution of cancer of Site A with respect to Attribute B were calculated separately for the various categories of Attribute C making allowance for age and sex variations and then these expected numbers were added together. Adjustments were made for more than one attribute by an extension of this process.

*Data on soils.*

The lettering and numbering of soil series used in the original "six-inch" maps is retained. Thus series having a common parent material geologically, are all indicated by the same capital letter, e.g., all derived from non-calcareous shales of Ordovician age are indicated P. The freely drained soil formed on this material weathered *in situ* is the P series. Further modifications are then shown by adding subscripts to P. Where the freely drained soil is on glacial drift (of the same material) it is called the $P_1$ series, if there is impedance of drainage evident in the subsoil it is the $P_{2a}$ series, when impedance shows to the surface and the soil is seasonally very wet it is the $P_2$ series. Occasionally soil wet at all seasons is encountered giving the $P_3$ series.

The first examination of cancer and soil series data suggested the desirability of combining some of the soil series together. This was best achieved by cutting the succession of soil series for each parent material at two points to form three groups.

At one end of the sequence were those soils subject to late spring and summer drought by virtue of shallowness or excessive drainage, in the middle those soils which because of the equable distribution of moisture in the profile at all seasons had a continuous period of plant growth and biological activity, at the other end those soils with a prolonged wet season by virtue of poor drainage. These are designated the $\alpha$, $\beta$ and $\gamma$ groups respectively. Table II shows the division into groups used for soils of this investigation, and also shows the dominant soil parent materials.

TABLE II.—*Distribution of Soil Series using the Series Letters, etc., from the "Six-inch" Soil Map, showing also the Dominant Parent Material.*

| | Dominant parent materials. | $\alpha$ soil group. | $\beta$ soil group. | $\gamma$ soil group. |
|---|---|---|---|---|
| A | Schists of the Mona Complex | $A_1$ rocky, $A_1$ thin, $A_1$ sandy, $A_1B_1$, A, $A_1$. | $A_1$–$A_{2a}$, $A_{2a}$, $A_{2a}$–$A_2$, $A_1P_1$, $A_2P_{2a}$, $A_1G_1$, $A_{2a}$ sandy. | $A_2$, $A_2$–$P_2$, $A_3$ |
| B | Acid igneous rocks and quartzite | $B_1$ rocky, B, $B_1$ | $B_1P_1$, $B_{2a}$, $B_1P_{2a}$ | $B_2$ |
| G | Carboniferous Limestone and other calcareous sediments | G + $G_1$ thin, $G_1$ sandy, $G_1$H. | $G_1$, E | $G_{2a}$ |
| P | Non-calcareous shales of Ordovician age | — | $P_1$, $P_1$–$P_{2a}$, $P_{2a}$ | $P_2$–$P_{2a}$, $P_2$. |
| S | Triassic sandstones and marls | — | $S_1$ | $S_{2a}$, $S_2$ |
| | Remainder | Blown sand, quartzite, $N_1$. | $M_1$ $N_1$–$N_{2a}$ | Alluvium, $N_2$–$N_{2a}$, $N_2$. |

*Soil and cancer site.*

Table III gives the observed and expected distributions of the total cancer deaths for the major cancer sites in each of the three soil groups. For only two is there a significant departure from expectation :

(a) *Cancer of the stomach* where there is a relative excess of deaths seen on $\beta$ soils and a deficiency on soils $\alpha$ and $\gamma$.

(b) *Cancer of the breast* where there is an excess on $\alpha$ soils and a deficiency on $\beta$ and $\gamma$ soils.

TABLE III.—*Cancer of Major Sites and Soil Group.*

| Cancer site. | | Soil. $\alpha$. | $\beta$. | $\gamma$. | $\chi^2$. | Probability ($n$ = 2). |
|---|---|---|---|---|---|---|
| Oesophagus | Observed | 18 | 20 | 9 | 0·91 | 0·7> P >0·5 |
| | Expected | 20·3 | 19·7 | 7·0 | | |
| Stomach | O. | 115 | 165 | 38 | 16·07 | P <0·001 |
| | E. | 136·8 | 134·4 | 46·8 | | |
| Intestines except rectum | O. | 64 | 66 | 26 | 0·60 | 0·8> P >0·7 |
| | E. | 67·2 | 65·8 | 23·1 | | |
| Rectum | O. | 29 | 35 | 9 | 1·22 | 0·7> P >0·5 |
| | E. | 31·0 | 31·2 | 10·9 | | |
| Other digestive system | O. | 37 | 32 | 11 | 0·35 | 0·9> P >0·8 |
| | E. | 34·5 | 33·7 | 11·8 | | |
| Lung, bronchus and trachea | O. | 25 | 19 | 13 | 3·47 | 0·20> P >0·10 |
| | E. | 24·6 | 24·5 | 8·0 | | |
| Breast | O. | 56 | 27 | 9 | 9·29 | 0·01> P >0·001 |
| | E. | 42·1 | 36·0 | 14·0 | | |
| Uterus | O. | 42 | 25 | 13 | 2·10 | 0·5> P >0·2 |
| | E. | 36·5 | 30·9 | 12·6 | | |
| All other sites | O. | 112 | 91 | 42 | 3·90 | 0·2> P >0·1 |
| | E. | 105·3 | 103·9 | 35·9 | | |
| All sites | | 498 | 480 | 170 | | |

*Social class.*

Social class was determined by the Registrar-General's 1950 Classification of Occupations, that of each married woman by the occupation of the husband. Persons described as of " independent means " and " no occupation " were allotted to Classes I–II and IV–V respectively. Expected values were calculated on the assumption that cancer of any given site was not associated differently with social class from cancer of all sites, allowance being made as before for age and sex. The results appear in Table IV. Cancer of the stomach was the only

TABLE IV.—*Cancer of Major Sites and Social Class (Registrar-General's classification).*

| Cancer site. | | Social class. I–II. | III. | IV–V. | $\chi^2$. | Probability ($n$ = 2). |
|---|---|---|---|---|---|---|
| Oesophagus | O. | 16 | 13 | 18 | 0·25 | 0·9> P >0·8 |
| | E. | 17·2 | 13·3 | 16·4 | | |
| Stomach | O. | 109 | 74 | 135 | 11·07 | 0·01> P >0·001 |
| | E. | 115·4 | 90·4 | 112·1 | | |
| Intestines except rectum | O. | 62 | 41 | 53 | 0·82 | 0·7> P >0·5 |
| | E. | 57·1 | 44·2 | 54·8 | | |
| Rectum | O. | 27 | 23 | 23 | 0·50 | 0·8> P >0·7 |
| | E. | 26·5 | 20·9 | 25·6 | | |
| Other intestinal | O. | 33 | 27 | 20 | 3·79 | 0·2> P >0·1 |
| | E. | 29·0 | 22·9 | 28·1 | | |
| Lung and bronchus | O. | 20 | 22 | 15 | 3·33 | 0·2> P >0·1 |
| | E. | 19·8 | 16·7 | 20·5 | | |
| Breast | O. | 30 | 33 | 29 | 1·55 | 0·5> P >0·3 |
| | E. | 33·1 | 27·8 | 31·2 | | |
| Uterus | O. | 25 | 28 | 27 | 0·56 | 0·8> P >0·7 |
| | E. | 27·9 | 25·7 | 26·4 | | |
| All other sites | O. | 92 | 71 | 82 | 0·61 | 0·8> P >0·7 |
| | E. | 87·9 | 70·1 | 86·9 | | |
| All sites | | 414 | 332 | 402 | | |

form of cancer which showed a significant deviation of observed from expected values using this classification.

In Anglesey a large proportion of the population is engaged in agriculture or allied work. There is a very wide economic range among these people though the average farm is small. The inclusion of many of this large group in social Class II was therefore open to question. These considerations and our special interest being in the association between cancer of the stomach and soil, suggested that it might be advisable to treat separately those occupations which bring the person into intimate connection with the land. The grouping eventually adopted was fourfold : a group "L", consisting of agricultural and allied occupations coded by the Registrar-General as 010–040 and 861, and the remainder of Social Classes I + II, III, IV + V. (To avoid confusion the classes so modified will be referred to as social groups.) Table V gives the results using this modified social grouping.

TABLE V.—*Cancer of Major Sites and Social Group* (*modified classification*).

| | | Social Group. | | | | | |
|---|---|---|---|---|---|---|---|
| Cancer site. | | I–II.* | III.† | IV–V.‡ | L. | $\chi^2$. | Probability ($n$ = 3). |
| Oesophagus | O. | 8 | 13 | 14 | 12 | 0·16 | 0·99> P >0·98 |
| | E. | 8·7 | 13·0 | 13·1 | 12·2 | | |
| Stomach | O. | 39 | 70 | 109 | 100 | 23·63 | P <0·001 |
| | E. | 57·6 | 87·6 | 89·1 | 83·8 | | |
| Intestines except rectum | E. | 34 | 41 | 43 | 38 | 1·27 | 0·8> P >0·7 |
| | O. | 29·0 | 42·8 | 43·9 | 40·4 | | |
| Rectum | O. | 13 | 23 | 17 | 20 | 1·05 | 0·8> P >0·7 |
| | E. | 13·2 | 20·2 | 20·4 | 19·3 | | |
| Other digestive tract | O. | 15 | 27 | 16 | 22 | 3·18 | 0·5> P >0·3 |
| | E. | 14·7 | 22·2 | 22·4 | 20·7 | | |
| Lung and bronchus | O. | 15 | 22 | 11 | 9 | 9·96 | 0·02> P >0·01 |
| | E. | 9·5 | 16·2 | 16·1 | 15·3 | | |
| Breast | O. | 23 | 31 | 25 | 13 | 5·24 | 0·2> P >0·1 |
| | E. | 18·3 | 26·8 | 26·1 | 20·8 | | |
| Uterus | O. | 15 | 27 | 22 | 16 | 0·38 | 0·95> P >0·90 |
| | E. | 16·1 | 24·6 | 22·7 | 16·5 | | |
| All other sites | O. | 48 | 69 | 65 | 63 | 0·82 | 0·9> P >0·8 |
| | E. | 42·9 | 69·8 | 63·9 | 63·9 | | |
| All sites | | 210 | 323 | 322 | 293 | | |

* Registrar-General's Classes I and II less occupation groups 010, 011, 020.
† Registrar-General's Class III less occupation groups 013, 014, 022, 030, 861.
‡ Registrar-General's Classes IV and V less occupation groups 012, 015, 019, 021, 029.

Cancer of the stomach shows a highly significant association with social group. In cancer of the lung and bronchus also the distribution is not likely to be a random one. The trend in breast cancer is of interest in that it is opposite to that in cancer of the stomach even though the departure from expectation does not reach conventional levels of significance.

*Water supply.*

The evidence for a relationship between soil and cancer site suggested examination for an association of cancer with the kind of domestic water supply. Data permitted only a two-fold grouping, (1) public mains, (2) other sources (wells, springs, stored rain-water).

The public mains are supplied for the most part from surface water such as small lakes fed by streams and springs. There is an exception where two small centres get their water from deep bore holes but the populations are too small to be separated in the analysis. The distinction between mains water and "other sources" is largely a matter of pollution rather than origin; the public main supplies are treated and controlled, whereas "other sources" are subject to occasional pollution.

Table VI is similar in construction to Tables III to V and compares the observed and expected distribution of deaths by site for the two categories of water supply. Cancer of the stomach is the only one to depart significantly from expectation, showing a relative deficiency of cases for public mains and an excess for "other sources". Cancer of the breast shows a tendency towards the reverse association though not quite reaching significance.

TABLE VI.—*Cancer of Major Sites and Water Supply.*

| Cancer site. | | Water supply. Public mains. | Other sources. | $\chi^2$. | Probability ($n$ = 1). |
|---|---|---|---|---|---|
| Oesophagus | O. | 19 | 28 | 0·10 | 0·8 > P >0·7 |
| | E. | 20·0 | 26·9 | | |
| Stomach | O. | 110 | 208 | 10·45 | 0·01> P >0·001 |
| | E. | 134·2 | 183·9 | | |
| Intestines except rectum | O. | 72 | 84 | 0·90 | 0·5 > P >0·3 |
| | E. | 66·6 | 89·5 | | |
| Rectum | O. | 33 | 40 | 0·38 | 0·7 > P >0·5 |
| | E. | 30·5 | 42·5 | | |
| Other intestinal | O. | 33 | 47 | 0·05 | 0·9 > P >0·8 |
| | E. | 33·9 | 46·1 | | |
| Lung, etc. | O. | 26 | 31 | 0·76 | 0·5 > P >0·3 |
| | E. | 22·9 | 34·2 | | |
| Breast | O. | 48 | 44 | 3·05 | 0·1 > P >0·05 |
| | E. | 40·0 | 51·9 | | |
| Uterus | O. | 34 | 46 | 0·002 | 0·98> P >0·95 |
| | E. | 33·8 | 46·2 | | |
| All other sites | O. | 107 | 138 | 0·95 | 0·5 > P >0·3 |
| | E. | 100·3 | 144·6 | | |
| All sites | | 482 | 666 | | |

In view of the association shown by cancer of the stomach and cancer of the breast with soil and with social group, the relationship between these two types of cancer and water supply was re-examined making allowance for variations arising from soil and social group in the two kinds of water supply. The adjusted distribution of expected values is shown in Table VII. When allowance is made for social group and soil, the association between cancer of the stomach and cancer of the breast and water supply is no longer evident.

TABLE VII.—*Cancer Site and Water Supply (adjusted for soil and social group).*

| Site | | Public Mains. | Other sources. | $\chi^2$. | Probability. ($n$ = 1) |
|---|---|---|---|---|---|
| Stomach: | O. | 110 | 208 | 1·90 | 0·20> P >0·10 |
| | E. | 120·2 | 197·8 | | |
| Breast (female deaths only): | O. | 48 | 43 | 0·28 | 0·70> P >0·50 |
| | E. | 45·3 | 45·7 | | |
| All sites (female deaths | | 255 | 323 | | |

*Soil and cancer of the stomach.*

A similar process of adjustment was applied to the association between cancer of the stomach and soil to allow for differences in both social group and water supply distributions. The results are shown in Table VIII.

The relative excess of cancer of the stomach on $\beta$ soil which was found for the unadjusted data (Table III) is still evident, though manifest mainly at ages of 65 years and over.

TABLE VIII.—*Cancer of the Stomach. Distributions by Soil (adjusted for social group, water supply, age and sex).*

| | Soil. | | | | |
|---|---|---|---|---|---|
| | $\alpha$. | $\beta$. | $\gamma$. | $\chi^2$. | Probability ($n = 2$) |
| Both sexes (all ages)— | | | | | |
| Stomach : O. . | 115 | 165 | 38 | 9·63 | $0·01 > P > 0·001$ |
| E. . | 129·4 | 142·0 | 46·6 | | |
| All sites . . . | 498 | 480 | 170 | | |
| Both sexes (age up to 65 years)— | | | | | |
| Stomach : O. . | 50 | 54 | 13 | 0·51 | $0·8 > P > 0·7$ |
| E. . | 50·2 | 51·7 | 15·1 | | |
| All sites . . | 214 | 188 | 66 | | |
| Both sexes (ages 65 and over)— | | | | | |
| Stomach : O. . | 65 | 111 | 25 | 12·32 | $0·01 > P > 0·001$ |
| E. . | 79·2 | 90·3 | 31·5 | | |
| All sites . . . | 284 | 292 | 104 | | |

In Table IX the social groups and the sexes have been considered separately, but as $\alpha$ and $\gamma$ soils showed deviations in the same direction these two are combined. From this it is seen that the association with $\beta$ soil is shown by both sexes. The social groups, however, show certain interesting differences. In Group L there does not appear to be any association with $\beta$ soil. There is a relative excess of deaths in the other three groups although only in Group III is this undoubtedly significant in the statistical sense.

*Soil and cancer of the breast.*

When corrections are applied for social group and water supply it is found that the association between cancer of the breast and $\alpha$ soil as compared with all other forms of cancer is still present (Table X). When the social groups are considered separately it is found that Group L does not show this association to the extent that it is shown by the others.

*Soil parent material.*

The nature of the parent materials of Anglesey soils has been referred to earlier. For further examination six groups were made according to the substances dominant in the parent material :

Group A : Schists of the Mona Complex.
Group B : Acid igneous rocks and quartzite.
Group G : Carboniferous Limestone and other calcareous sediments.
Group P : Non-calcareous shales of Ordovician age.
Group S : Triassic sandstones and marls.
Remainder : Blown sand, marine and river alluvium, etc. (Table II).

TABLE IX.—*Cancer of the Stomach—Distribution by Soil (Adjusted for age, Sex and Water Supply) Social Groups considered separately.*

| | | Soil: β. | Soil: α plus γ. | $\chi^2$. | Probability ($n = 1$). |
|---|---|---|---|---|---|
| I–II* | | | | | |
| Stomach | O. | 18 | 21 | 2·43 | 0·20> P >0·1 |
| | E. | 13·8 | 25·2 | | |
| All sites | | 73 | 137 | | |
| III* | | | | | |
| Stomach | O. | 40 | 30 | 12·03 | <P 0·001 |
| | E. | 27·5 | 42·5 | | |
| All sites | | 119 | 204 | | |
| IV–V* | | | | | |
| Stomach | O. | 63 | 46 | 3·84 | P = 0·05 |
| | E. | 54·7 | 54·3 | | |
| All sites | | 152 | 170 | | |
| L. | | | | | |
| Stomach | O. | 44 | 56 | 0·25 | 0·70> P >0·50 |
| | E. | 46·0 | 54·0 | | |
| All sites | | 136 | 157 | | |
| All social groups : | | | | | |
| Males | | | | | |
| Stomach | O. | 94 | 84 | 5·87 | 0·02> P >0·01 |
| | E. | 82·3 | 95·7 | | |
| All sites | | 251 | 319 | | |
| Females | | | | | |
| Stomach | O. | 71 | 69 | 4·98 | 0·05> P >0·02 |
| | E. | 59·7 | 80·3 | | |
| All sites | | 229 | 249 | | |

* See note beneath Table V.

TABLE X.—*Cancer of the Breast (Female Deaths only)—Distribution by Soil Group (adjusted for age, social group and water supply groups).*

| | Soil: α. | Soil: β. | Soil: γ. | $\chi^2$. | Probability ($n = 2$). |
|---|---|---|---|---|---|
| All social groups | | | | | |
| Breast : O. | 56 | 27 | 8 | 8·14 | 0·02> P >0·01 |
| E. | 43·9 | 33·9 | 13·3 | | |
| All sites | 264 | 227 | 87 | | |
| Social groups except L.— | | | | | |
| Breast : O. | 50 | 22 | 7 | 8·62 | 0·02> P >0·01 |
| E. | 38·3 | 29·4 | 11·4 | | |
| All sites | 210 | 172 | 67 | | |
| Social group L.— | | | | | |
| Breast : O. | 6 | 5 | 1 | 0·06* | 0·9> P >0·8 |
| E. | 5·6 | 4·5 | 1·9 | | |
| All sites | 54 | 55 | 20 | | |

* When β plus γ is compared with α ($n = 1$).

The association between cancer of the stomach and β soil has already been shown. To see if this association was influenced by the geological nature of the soil parent material the distribution of cancer of the stomach by parent material was compared with the expected distribution after allowance had been made for social group and soil (Table XIA). Cancer of the stomach is commoner in Group P. The result of further examination for $\beta$ soil separate from $\alpha$ plus $\gamma$ is shown in Tables XIB and XIC. The association of cancer of the stomach with group P parent material is evident only on $\beta$ soils.

A similar calculation was applied to the female deaths from cancer of the breast and Table XID gives the distribution of these deaths by soil parent material, compared with the expected distribution after adjusting for social group and soil. The numbers in the categories other than A are small but the agreement with expectation appears to be reasonably good.

TABLE XIA.—*Cancer of the Stomach—Soil Parent Material (adjusted for social group and soil).*

| | Soil parent material | | | | | | |
|---|---|---|---|---|---|---|---|
| | A. | B. | G. | P. | S. | R. | |
| Stomach : O. | 207 | 12 | 31 | 27 | 32 | 9 | 318 |
| E. | 220·6 | 15·5 | 25·5 | 18·7 | 29·6 | 8·2 | 318·1 |
| All sites | 805 | 55 | 75 | 58 | 116 | 39 | 1148 |

If P soil is compared with the remainder, $n = 1$, $\chi^2 = 5\cdot75$ and probability is $0\cdot02 > P > 0\cdot01$.

TABLE XIB.—*Cancer of the Stomach—Soil Parent Material—$\beta$ Soil (adjusted for social group).*

| | Soil parent material. | | | | | | |
|---|---|---|---|---|---|---|---|
| | A. | B. | G. | P. | S. | R. | |
| Stomach : O. | 99 | 1 | 25 | 21 | 16 | 3 | 165 |
| E. | 112·4 | 1·9 | 19·8 | 14·1 | 14·8 | 2·0 | 165 |
| All sites | 328 | 5 | 55 | 43 | 43 | 6 | 480 |

If A and B, S and R figures are combined, $n = 3$, $\chi^2 = 10\cdot32$ and probability is $0\cdot02 > P < 0\cdot01$.

TABLE XIC.—*Cancer of the Stomach—Soil Parent Material $\alpha$ plus $\gamma$ Soil Group (adjusted for social group).*

| | Soil parent material. | | | | | | |
|---|---|---|---|---|---|---|---|
| | A. | B. | G. | P. | S. | R. | |
| Stomach : O. | 108 | 11 | 6 | 6 | 16 | 6 | 153 |
| E. | 108·2 | 13·6 | 5·7 | 4·8 | 14·8 | 6·2 | 153·3 |
| All sites | 477 | 50 | 20 | 15 | 73 | 33 | 668 |

$\chi^2 = 1\cdot31$ ; $n = 5$ ; $0\cdot95 > P > 0\cdot90$.

TABLE XID.—*Cancer of the Breast (Female Deaths only)—Soil Parent Material (adjusted for social group and soil).*

| | Soil parent material. | | | | | | |
|---|---|---|---|---|---|---|---|
| | A. | B. | G. | P. | S. | R. | |
| Breast : O. | 73 | 6 | 3 | 1 | 6 | 2 | 91 |
| E. | 68·9 | 4·7 | 4·2 | 3·1 | 6·8 | 3·3 | 91 |
| All sites | 414 | 28 | 28 | 26 | 61 | 21 | 578 |

$\chi^2$ not calculated.

### CONCLUSION AND SUMMARY.

Fatal cases of cancer, occurring in Anglesey during ten years, have been examined with respect to the soil at the home of each deceased person, the domestic water supply, the social class or group, and the geology of the soil parent material.

The analysis of mortality from cancer of particular sites was made in comparison with that for cancer of all sites taken together. It was not possible to relate cancer mortality to the populations at risk living on the different soil groups in the different social classes, etc.

Cancer of the stomach and cancer of the breast were each found to be associated with a group of soil series. Associations were also demonstrated for these two cancer sites with respect to water supply and social class or group. Cancer of the lung and bronchus showed some association with social group.

The association with soil, in the case of stomach and breast, was independent of social group and water supply.

For cancer of the stomach the association with soil was shown by each sex but was statistically significant only for those aged 65 and over.

The Social Group L, of persons having close connection with the land, did not show this association with soil group either for cancer of the stomach or cancer of the breast.

When allowance was made for social class and soil, association between cancer of the stomach and the soil parent material was shown only by the Geological Group P (mainly non-calcareous Ordovician shales).

The kinds of soil which are positively associated with cancer of the stomach (referred to as $\beta$ soils) show great biological activity over a long season. In general $\beta$ soils have a high content of organic matter but the converse is not necessarily true since high content of organic matter—and "loss on ignition" which is often wrongly used to measure it—may arise from a great variety of conditions.

The chain of soil series on each parent material has been divided at two points in a fashion that fits Anglesey. It would be dangerous to assume that division would occur at the same points in other counties. Allowance, requiring specialist local knowledge, would be needed for differences in altitude, topography, aspect and climate in order to arrive at the proper grouping of $\alpha$, $\beta$, and $\gamma$ soils.

The authors are grateful to Dr. Percy Stocks, C.M.G., M.D., F.R.C.P., and to Dr. F. Smithson, D.Sc., F.G.S., for their helpful advice, encouragement and kindly criticism.